# Effects of *Arthrospira platensis* on Human Umbilical Vein Endothelial Cells

**DOI:** 10.3390/life14101253

**Published:** 2024-10-01

**Authors:** Anne Krüger-Genge, Kudor Harb, Steffen Braune, Conrad H. G. Jung, Sophia Westphal, Stefanie Bär, Olivia Mauger, Jan-Heiner Küpper, Friedrich Jung

**Affiliations:** 1 Life Science and Bioprocesses, Fraunhofer Institute for Applied Polymer Research (IAP), 14476 Potsdam, Germany; harbkudor@outlook.de (K.H.); sophia.westphal@iap.fraunhofer.de (S.W.); stefanie.baer@iap.fraunhofer.de (S.B.); o.mauger@web.de (O.M.); 2 Institute of Biotechnology, Molecular Cell Biology, Brandenburg University of Technology Cottbus-Senftenberg, 01968 Senftenberg, Germanykuepper@b-tu.de (J.-H.K.); friedrich.jung@b-tu.de (F.J.); 3 Faculty of Health Sciences Brandenburg, Brandenburg University of Technology Cottbus-Senftenberg, 01968 Senftenberg, Germany; 4 Carbon Biotech, Social Enterprise Stiftungs AG, 01968 Senftenberg, Germany

**Keywords:** human umbilical vein endothelial cells, HUVEC, *Arthrospira platensis*

## Abstract

Atherosclerosis is initiated by injury or damage to the vascular endothelial cell monolayer. Therefore, the early repair of the damaged vascular endothelium by a proliferation of neighbouring endothelial cells is important to prevent atherosclerosis and thrombotic events. *Arthrospira platensis* (AP) has been used as a dietary supplement, mainly due to its high content of vitamins, minerals, amino acids, and pigments such as chlorophylls, carotenoids, and phycocyanin, ingredients with antioxidant, anti-inflammatory, and anti-thrombotic properties. Therefore, in this prospective, placebo-controlled, data-driven, sample-size-estimated in vitro study, we tested whether an aqueous extract of AP at different concentrations (50, 100, and 200 µg/mL) had an effect on the different cellular parameters of human umbilical vein endothelial cells. Therefore, cell impedance measurement and cell proliferation were measured to investigate the monolayer formation. In addition, cell viability, integrity, and metabolism were analysed to evaluate singular cellular functions, especially the antithrombotic state. Furthermore, cell–cell and cell–substrate interactions were observed. The highest proliferation was achieved after the addition of 100 µg/mL. This was consistently confirmed by two independent optical experiments in cell cultures 48 h and 85 h after seeding and additionally by an indirect test. At this concentration, the activation or dysfunction of HUVECs was completely prevented, as confirmed by prostacyclin and interleukin-6 levels. In conclusion, in this study, AP induced a significant increase in HUVEC proliferation without inducing an inflammatory response but altered the hemostasiological balance in favour of prostacyclin over thromboxane, thereby creating an antithrombotic state. Thus, APE could be applied in the future as an accelerator of endothelial cell proliferation after, e.g., stent placement or atherosclerosis.

## 1. Introduction

The basal state of the endothelial cell (EC) surface in the cardiovascular system is thought to be essentially anticoagulant or non-thrombogenic, which is related through the expression of specific receptors and the release of cell mediators [1,2]. However, biologicals, drugs, increased shear forces, or the placement of vascular stents can induce an activated or perturbed state of the EC, ranging from morphological abnormalities to cell detachment. This can lead to the exposure of the vascular basement membrane, thereby providing a site where the contact system of blood coagulation could be activated with subsequent thrombus formation. As a result, a reduced blood supply occurs in the downstream tissue. To prevent platelet aggregation/adhesion the vascular endothelium has an anti-thrombogenic potency by expressing anticoagulant substances including prostacyclin and nitric oxide [3,4,5]. According to Russell Ross’s “response to injury theory”, atherosclerosis is initiated by damage to the vascular endothelial cell monolayer [6,7]. The early repair of damaged vascular endothelium by the proliferation of neighbouring endothelial cells is important to prevent atherosclerosis and thrombotic events. 

The blue–green alga *Arthrospira platensis* (AP), commonly known as spirulina, has been used as a dietary supplement for a long time mainly due to its high content of vitamins, minerals, amino acids, and pigments such as chlorophylls, carotenoids, and phycocyanin [8,9], ingredients with antioxidant, anti-inflammatory, and anti-thrombotic properties [10,11,12]. AP also has great potential as a supplement to the human diet, particularly due to its high protein content of up to 70% [13,14,15]. Recent studies have also demonstrated the first beneficial effects on endothelial cells and platelets [16].

**Figure 1:**
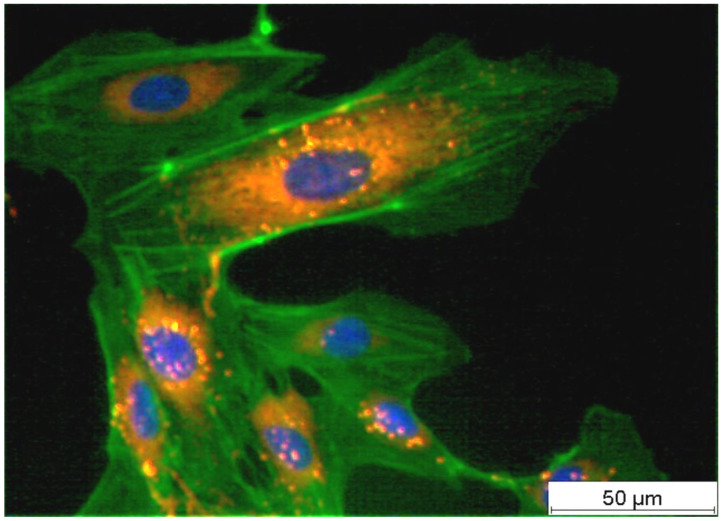
Threefold staining of adherent HUVECs on a tissue culture plate two days after seeding (cell nucleus in blue, actin stress fibres in green, and von Willebrand factor in red).

Figure 1 shows a typical example of HUVECs on a polystyrene tissue culture plate surface two days after seeding. HUVEC actin fibres, cell nuclei, and von Willebrand Factor (vWF) were stained by immunocytochemistry. All the cells contained vWF, allowing them to be characterised as endothelial cells.

In a first preliminary pilot study, it was shown that the supplementation of human umbilical vein endothelial cells (HUVECs) with an aqueous extract of AP (APE) did not harm the cells but dose-dependently enhanced the development of an endothelial cell monolayer in vitro four days after seeding [17]. In addition, some studies reported that the concurrent use of AP might improve the formation of thrombotic events in patients with COVID-19 [18,19,20].

These data led us to hypothesise that APE can influence the proliferation of HUVECs and the formation of thrombi. Therefore, in a prospective, placebo-controlled, in vitro study, we tested whether an aqueous AP extract at different concentrations (50, 100, and 200 µg/mL) had an effect on the proliferation, adherence, metabolism, viability, cell membrane integrity of HUVECs, and parameters playing a role in the process of thrombus formation.

## 2. Material and Methods

### 2.1. Study Design

The in vitro study was performed in a prospective, placebo-controlled design using primary human umbilical vein endothelial cells (HUVECs). The HUVEC density at baseline was 3500 (xCELLigence system E-plate) and 15,000 HUVECs/well (24-well format). The development of a HUVEC monolayer on tissue culture plates (TCPs) was evaluated over 85 h. The study was performed with negative and positive controls (HUVEC lysed with Triton X-100, West Chester, PA, USA). According to a data-driven sample size estimation, all experiments were performed in eight independent replicates with two repetitions each.

HUVECs were seeded in well plates in a cell culture medium supplemented with APE at three different concentrations (50, 100, and 200 µg/mL) [17].

The following cellular parameters were investigated: cell adherence, proliferation, viability, outer membrane integrity, cell metabolism, cell function, cell–cell contacts, and stress fibre formation. 

The HUVECs were evaluated at least 12 h after APE and cell culture medium supplementation to avoid a misinterpretation of the morphological state of the endothelial cells [21]. 

### 2.2. Endothelial Cells

HUVECs were purchased from Lonza (Basel, Switzerland). The HUVECs were cultivated under static conditions in a standard humidified incubator at 37 °C with 5% CO_2_ supplemented with 100 U/mL penicillin/streptomycin, and 10% foetal calf serum (Thermo Fisher Scientific, Vienna, Austria; 10270106). Cell experiments were performed using HUVECs in passage 4 [22]. The cell culture medium (Lonza, Basel, Switzerland) was changed every second day.

### 2.3. Real-Time Measurement of the HUVEC Monolayer Formation 

The effect of APE on the proliferation of HUVECs was assessed in real-time by the xCELLigence system E-plate 16 (ACEA Biosciences, Inc., San Diego, CA, USA) for 85 h at 10 min intervals. Details of the methods have already been published formerly [17].

The xCELLigence system consists of a plate station with E-plates in a 96-well-plate format and software for automatic and real-time data acquisition and display. The system measures electrical impedance across micro-electrodes integrated on the bottom of tissue culture E-plates. It allows one to monitor changes in the adherence, spreading, and proliferation of HUVECs in real-time based on the measured cell–electrode impedance [23]. From these data, a parameter termed “cell index (CI)” can be calculated as follows:

$$CI=\begin{array}{c} max\\ i=1,\ldots ,N \end{array}\;[\;(\frac{Rcell\left(fi\right)}{Rb(fi)}-1)]$$

where *R_b_*(*f*) and *R_cell_*(*f*) are the frequency-dependent electrode resistances (a component of the impedance) without cells or with cells present, respectively. N is the number of the frequency points at which the impedance is measured. Thus, cell index (CI) is a quantitative measure of the status of the cells in an electrode-containing well. The effect of APE in increasing concentrations (0 µg/mL, 50 µg/mL, 100 µg/mL, and 200 µg/mL) on the CI was measured. 

### 2.4. Fabrication of APE

AP bacteria were obtained from the “The Culture Collection of Algae at the Göttingen University” (strain: SAG21.99) and cultured in a polyethylene flat-type (2 cm) bioreactor. The growth medium (Zarrouk medium [24]) was initially sterilised at 121 °C in an HV-50 autoclave (SYSMEX VX-95, Sysmex, Norderstedt, Germany) for 15 min. 

The resulting AP powder was stirred overnight in a sterile 0.9% NaCl solution (B.Braun, Melsungen, Germany) at room temperature (10 mg/mL). The extracts were then centrifuged at 3400× *g* for 5 min, followed by filtration through 0.4 and 0.22 µm filters (TPP). The extracts were stored at 4 °C until further processing. 

### 2.5. Analysis of Cell Viability and Cell Density

Cell viability was analysed using fluorescein diacetate (FDA, 25 µg/mL Invitrogen, Carlsbad, CA, USA) and propidium iodide (PI, 2 µg/mL, Sigma, Taufkirchen, Germany). FDA stained vital cells in green, PI stained dead cell in red. Subsequently, three pictures per sample were taken in 10-fold primary magnifications (DMIL LED, Leica, Wetzlar, Germany). The HUVECs were stained 48 h and 85 h after cell seeding. The cell density was determined by cell counting three microscopic images per sample.

### 2.6. Analysis of Cell Metabolism

The metabolic activity of the HUVECs was investigated using an MTS assay (Cell Proliferation Assay, Promega, Madison, WI, USA). In this assay, cytosolic NADH-dependent dehydrogenases of metabolically active cells reduce the tetrazol group of added components to formazan. The amount of formazan is correlated with the number of viable cells.

### 2.7. Cell Membrane Integrity

Cell membrane integrity was analysed using an LDH-Cytotoxicity Assay Kit II © (BioVision Inc., Milpitas, CA, USA). The release of intracellular lactate dehydrogenase in the cell culture supernatant was measured two and four days after cell seeding. 

### 2.8. Secretion Profile Screening

The release of Prostacyclin (PGl_2_) and Thromboxane A_2_ (TXA_2_) was measured as a sign of HUVEC function two and four days after cell seeding using ELISA. The concentrations of PGl_2_ and TXA_2_ were detected in the cell culture supernatant using the 6-keto Prostaglandin F1α EIA Kit (Cayman Chemicals, Hamburg, Germany) and theTXA2 Kit (Cloude-Clone Corp., Houston, TX, USA).

### 2.9. Staining of Cell–Cell and Cell–Substrate Contacts

Two and four days after cell seeding on glass coverslips, the HUVECs were washed with Phosphate-Buffered Saline, containing magnesium and calcium (PBS^+/+^; Biochrom GmbH, Berlin, Germany). Subsequently, the HUVECs were incubated with a 4% paraformaldehyde solution (C.Roth, Karlsruhe, Germany) followed by washing with PBS without magnesium and calcium (PBS^−/−^; Sigma Aldrich, St. Louis, MO, USA). For cell permeabilisation, the HUVECs were incubated with 0.25% Triton X-100 solution (VWR, Darmstadt, Germany) followed by blocking of unspecific binding sites with BSA solution (3% in PBS^−/−^, Biochrom GmbH, Berlin, Germany). vWF was stained using vWF polyclonal rabbit primary antibodies (1:500 in PBS, Thermo Fisher Scientific, Waltham, MA, USA), VE-cadherin was stained using anti-VE-cadherin rabbit antibodies (1:60 in PBS, Bejing, China), and vinculin was stained using vinculin mouse monoclonal antibodies (1:100 in PBS, Invitrogen, Carlsbad, CA, USA). After an incubation of 1.5 h, the HUVECs were washed with PBS. Thereafter, the cells were incubated with a Phalloidin-iFluor 488 reagent (1:500 in PBS with BSA, Abcam, Cambridge, UK) to stain cell cytoskeletons, anti-rabbit Alexa Fluor 555 (1:1000, Invitrogen, Carlsbad, CA, USA), and anti-mouse Alexa Fluor 488 (1:1000, Invitrogen, Carlsbad, CA, USA), followed by nuclei staining using 4‘,6-diamidino-2-phenylindole (DAPI, Roth, Karlsruhe, Germany). Finally, fixated HUVECs were washed with PBS and embedded using ProLong™ Gold Antifade Mountant (Invitrogen, Carlsbad, CA, USA).

### 2.10. Statistical Analysis

With the data of a previous pilot study [17], a sample size estimation was performed, and as a result, a sample size of n = 8 experiments was chosen for all experiments. The confirmatory aim parameter was the HUVEC density. Gaussian distribution was checked using the Kolmogorov–Smirnov test.

Mean values with standard deviations are given for all samples. Statistical analysis was performed using a one-way anova. Differences of *p* < 0.05 were considered significant.

## 3. Results

### 3.1. Effect of Arthrospira on the Number of Adherent HUVECs

**Figure 2:**
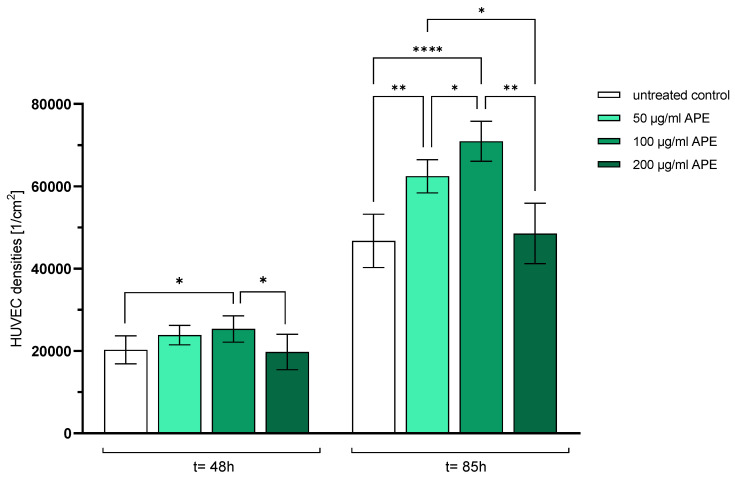
HUVEC densities (1/per cm^2^) 48 h and 85 h after seeding. The mean values with standard deviation over n = 8 independent tests are shown. *p* values: * = *p* ≤ 0.05, ** = *p* ≤ 0.01, **** = *p* ≤ 0.0001.

Figure 2 shows the densities of the HUVECs [1/cm^2^] 48 h and 85 h after seeding in 24-well plates. The endothelial cells reacted very differently to the addition of APE depending on the concentration of the extract.

Compared to the control cells (46,720.0 ± 6473.9), there was a significant increase in endothelial cells 85 h after the addition of APE at 50 µg/mL (62,428.3 ± 4035.8) and at 100 µg/mL (70,931.1 ± 4850.2). In contrast, the addition of 200 µg/mL did not affect the number of adherent HUVECs (48,519.6 ± 7348.7).

Figure 2 clearly shows that the addition of 100 µg/mL APE resulted in a significant increase in the HUVEC density already after 48 h (25%) and even more so after 85 h (52%) of culturing compared to that in the control cells. 

**Figure 3:**
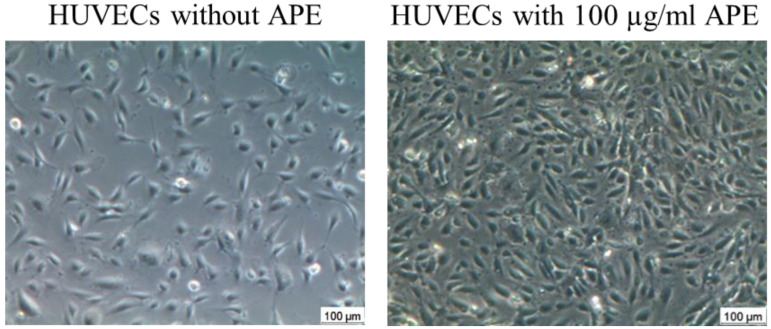
Representative examples of the HUVEC monolayers of the control culture (without APE) and the culture wit APE in the concentration of 100 µg/mL (85 h after seeding).

The significantly higher HUVEC density compared to that in the control culture is depicted in Figure 3.

**Figure 4:**
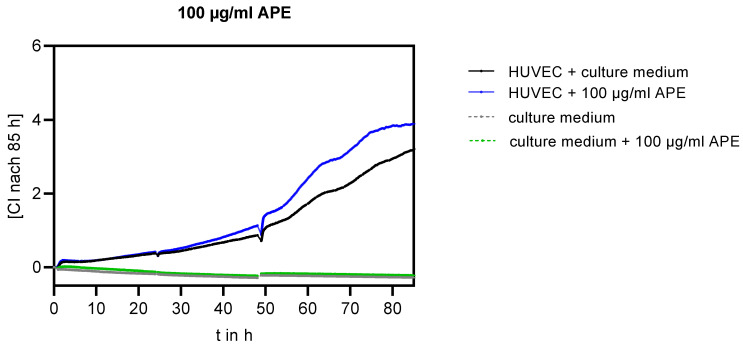
Continuous detection of the HUVEC monolayer for the addition of 100 µg/mL APE to the culture medium using the xCelligence system, n = 8 each.

In addition, the growth of the endothelial cell monolayer was analysed using a continuous method (cell index measurement by the real-time xCELLigence system [23]). As an example, the course of monolayer growth for the addition of 100 µg/mL APE is shown in Figure 4.

While the addition of APE at a concentration of 50 µg/mL caused no change in monolayer growth compared to that in the control cells (*p* = 0.60), the cell index was significantly increased by 22% 85 h after the addition of 100 µg/mL of APE compared to that in the control cells (*p* = 0.0054). In contrast, the cell index decreased by 14% after the addition of 200 µg/mL (see Table 1).

The independent continuous experiments on the growth of the endothelial cell monolayer confirmed the endothelial experiments of the first series of measurements, in which the growth of HUVECs was recorded discontinuously after two and four days.

### 3.2. Effect of Arthrospira on the Viability of Adherent HUVECs

**Figure 5:**
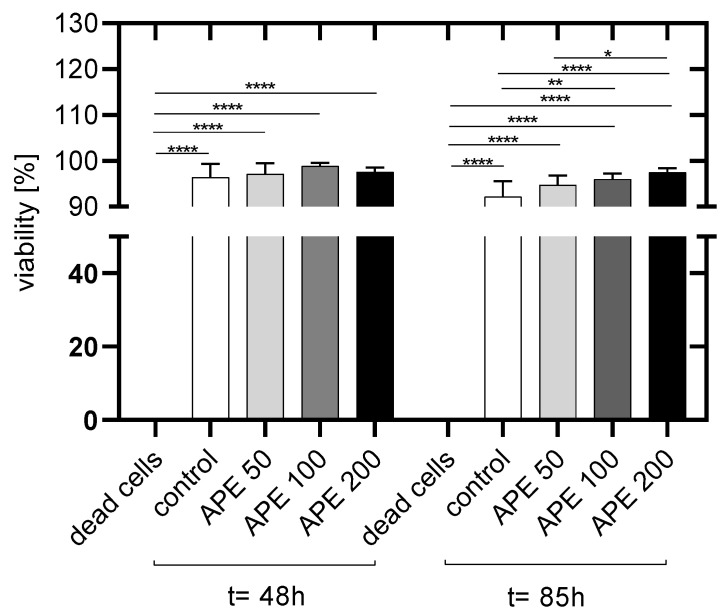
Viability of HUVECs [%] 48 h and 85 h after seeding (dead cells: HUVECs lysed with Triton X-100, control: untreated HUVECs in culture medium. APE 50: HUVECs in culture medium with 50 µg/mL AP extract. APE-100: HUVECs in culture medium with 100 µg/mL AP extract. APE-200: HUVECs in culture medium with 200 µg/mL APE. The mean values with standard deviation over n = 8 independent tests are shown. *p* values: * = *p* ≤ 0.05, ** = *p* ≤ 0.01, **** = *p* ≤ 0.0001.

Two days after cell seeding and continuous cultivation in culture medium with APE, no significant differences in viability between untreated and APE-treated HUVECs could be detected (Figure 5).

**Figure 6:**
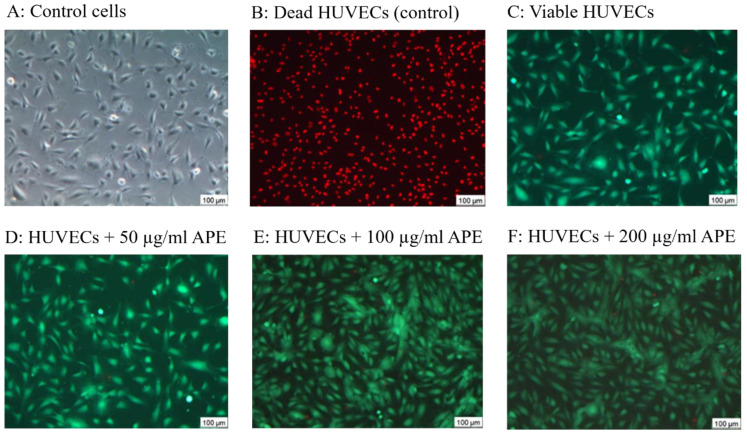
Representative images of HUVECs. (**A**) Phase contrast image; (**B**) dead HUVECs stained with PI in red, (**C**) FDA-stained HUVEC monolayer showing viable cells in green; (**D**) HUVEC monolayer supplemented with 50 µg/mL APE; (**E**) HUVEC monolayer supplemented with 100 µg/mL APE; (**F**) HUVEC monolayer supplemented with 200 µg/mL APE. Live (green)/dead (red) staining was imaged by fluorescence microscopy (10× primary magnification).

Figure 6 shows representative images of the HUVEC monolayers for the five sets of experiments versus the control HUVECs. The control cells are presented in phase contrast microscopy: dead cells are stained in red, and viable cells in green.

Eighty-five hours after cell seeding, a higher number of viable HUVECs cultured with APE-100 and APE-200 was detected compared to that in the control cells. There were also significantly more viable HUVECs after the addition of APE-200 than after the addition of AP 50.

The staining with PI showed that only very few dead cells were present. In addition, the fact that almost exclusively endothelial cells were present in the culture was also of decisive importance, as the extraction of the endothelial cells from the umbilical cord vein could introduce other vascular cells into the culture also [25]. 

### 3.3. Effect of Arthrospira platensis on the Integrity of the Outer Membrane of HUVECs

**Figure 7:**
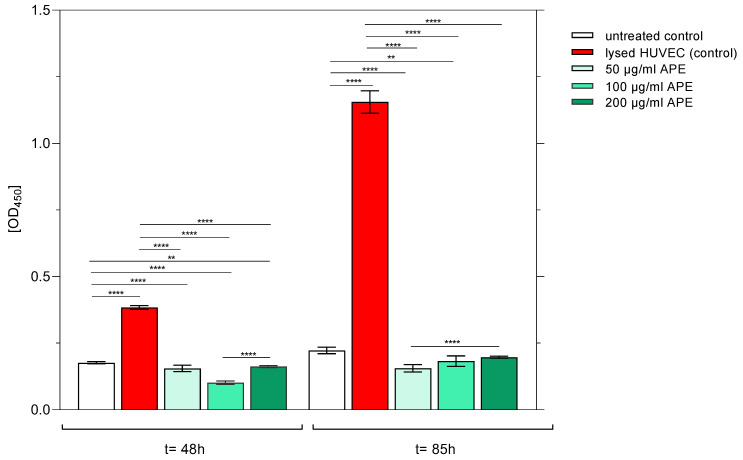
Extracellular lactate dehydrogenase (LDH) activity of HUVECs as an indicator of membrane integrity two and four days after cell seeding. Lysed HUVEC/positive control: HUVECs lysed with Triton X-100. Untreated control/negative control: HUVECs in culture medium. APE-50: HUVECs in culture medium with 50 µg/mL APE. AP-100: HUVECs in culture medium with 100 µg/mL APE extract. AP200: HUVECs in culture medium with 200 µg/mL APE extract. The mean values with standard deviation over n = 8 independent tests are shown. *p* values: ** = *p* ≤ 0.01, **** = *p* ≤ 0.0001.

To analyse the influence of the possible damage of APE on HUVECs, the integrity of the outer endothelial cell membrane was examined. The extent of damage was measured by the release of lactate dehydrogenase (LDH), normally found only in the cytosol, into the cell culture supernatant. The higher the release, the greater the extent of the cell damage. It was found that the highest release was measured in the cell culture supernatant of the untreated control cells. However, the level of LDH release was significantly lower compared to that in the positive control (lysed cells: HUVECs showing maximum LDH release) (Figure 7). Therefore, two days after cell seeding, no cell damage caused by the addition of the APE was detected. Interestingly, the lowest LDH release also occurred in HUVECs treated with APE-100.

Four days after cell seeding, LDH release was significantly lower in cell cultures treated with APE-50 and APE-100 compared to that in the untreated control. Cells cultured with APE-200 showed no differences compared to the control cells. It can therefore be concluded that the cultivation of cells with APE does not have a negative effect on the integrity of the cell membrane and does not induce cell damage in the concentration range investigated.

### 3.4. Arthrospira Induced a Concentration-Dependent Decrease in Metabolic Activity in HUVECs

The metabolic activity of the untreated HUVECs was determined by the MTS assay and taken as 100%. The absorbance values obtained for the APE-treated cells were then normalised to this value.

**Figure 8:**
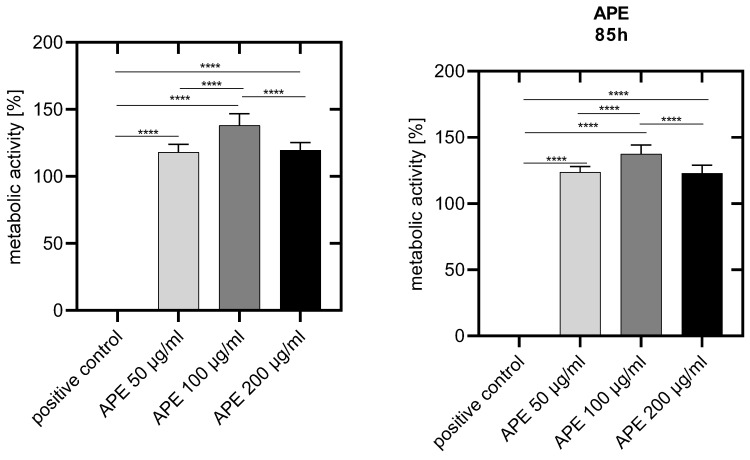
Metabolic activity of HUVECs two and four days after cell seeding. Positive control: HUVECs lysed with Triton X-100. APE-50: HUVECs in culture medium with 50 µg/mL APE. APE-100: HUVECs in culture medium with 100 µg/mL APE. APE-200: HUVECs in culture medium with 200 µg/mL APE. The mean values with standard deviation over n = 8 independent tests are shown. *p* values: **** = *p* ≤ 0.0001.

Forty-eight hours after cell seeding, the metabolic activity of all the APE-treated HUVECs was significantly higher than 100% and thus above that of the untreated control cells. A significantly increased metabolic activity was observed in cells cultured with 100 µg/mL APE compared to 50 and 200 µg/mL APE (Figure 8).

Eighty-five hours after cell seeding, the metabolic activity of all the APE-treated cells was still above 100% and thus significantly higher than that of the untreated control. The highest metabolic activity was again observed in HUVECs cultured with APE-100 (Figure 8).

### 3.5. Arthrospira Affects the HUVEC Function 

#### 3.5.1. Prostacyclin

Prostacyclin is an eicosanoid that regulates the diameter of blood vessels and inhibits platelet aggregation [26]. It is also considered a functional marker of endothelial cells. Excessive release of prostacyclin is considered as a sign of excessive stress on endothelial cells and is referred to as perturbation. Prostacyclin secretion after 48 and 85 h is shown below; the prostacyclin concentration was normalised to the number of HUVECs. 

**Figure 9:**
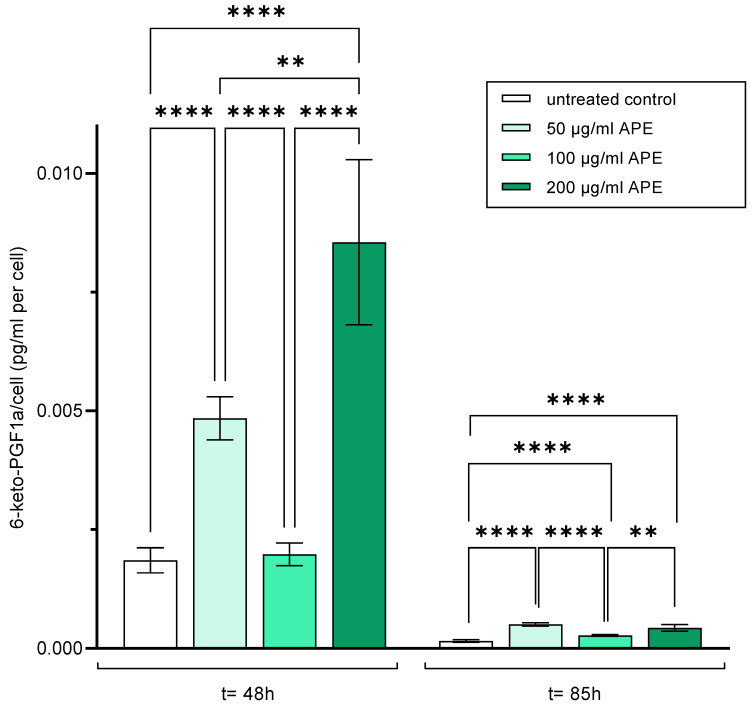
Prostacyclin (PGl2)—secretion of HUVECs two and four days after seeding (untreated control: HUVECs in culture medium; 50 µg/mL APE: HUVECs in culture medium with 50 µg/mL APE; 100 µg/mL APE: HUVECs in culture medium with 100 µg/mL APE; 200 µg/mL APE200: HUVECs in culture medium with 200 µg/mL APE. The mean values with standard deviation over n = 8 independent tests are shown. *p* values: ** = *p* ≤ 0.01, **** = *p* ≤ 0.0001.

Forty-eight hours after cell seeding, a significantly increased prostacyclin release was observed in APE-200-treated HUVECs compared to untreated and APE-50- and APE-100-treated cells (Figure 9). 

Eighty-five hours after cell seeding, prostacyclin release decreased significantly in all cells. The significantly lowest release was observed for untreated HUVECs. The significantly highest release was observed in APE-50-treated HUVECs compared to control and APE-100-treated cells. HUVECs cultured with APE-100 showed the significantly lowest prostacyclin release compared to APE-50- and APE-200-treated cells (Figure 9).

#### 3.5.2. Interleukin-6 

To investigate the influence of cultivating HUVECs with APE on a possible inflammatory activation of the cells, the release of the pro-inflammatory interleukin-6 (IL-6) was analysed. The IL-6 release was normalised to the respective number of HUVECs. 

**Figure 10:**
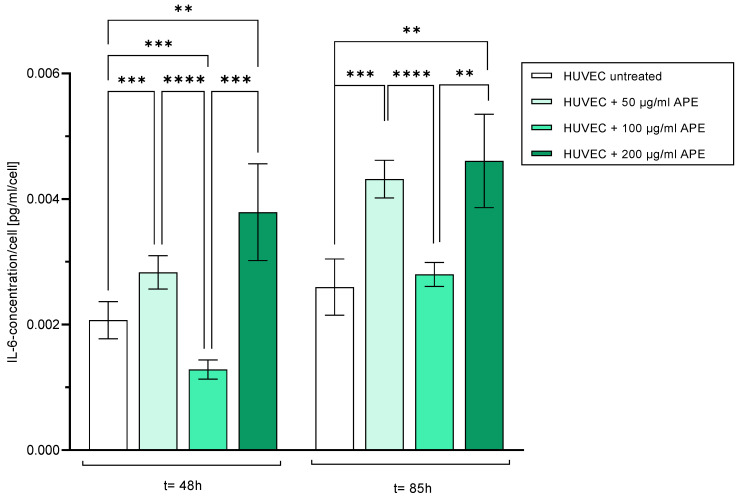
Interleukin-6 (IL-6) secretion of HUVECs 48 h and 85 h after cell seeding. Mean values with standard deviation over n = 8 independent tests are shown. *p* values: ** = *p* ≤ 0.01, *** = *p* ≤ 0.001, **** = *p* ≤ 0.0001.

Forty-eight hours after seeding, the significantly highest IL-6 release was found for HUVECs treated with APE-200 compared to those of the untreated and APE-100-treated cells. The lowest release was found in APE-100-treated HUVECs (Figure 10).

Eighty-five hours after cell seeding, IL-6 release increased in all samples. The lowest IL-6 release occurred in untreated control cells compared to those in APE-50- and APE-200-treated cells. The highest release was observed in APE-200-treated HUVECs compared to that in APE-100-treated and untreated cells. Compared to APE-200- and APE-50-treated cells, the release of IL-6 by APE-100-treated cells was significantly reduced (Figure 10). 

It can therefore be concluded that the release of IL-6 was significantly increased by culturing the cells in APE-50 and APE-200, whereas culturing the cells in APE-100 was comparable to the release of IL-6 from untreated HUVECs. Thus, there appears to be a concentration-dependent pro-inflammatory activation of the cells by cultivation in AP extract.

#### 3.5.3. Thromboxane 

Thromboxane (TXA2) causes an increase in vascular tone and thus a constriction of the blood vessels as well as an activation and aggregation of platelets. 

TXA2 was not detected in the cell culture supernatant of HUVECs cultured with AP extract.

There are two possible reasons for this finding: TXA2 was not released, or the amount of TXA2 released was below the detection limit of 15.625 pg/mL of the assay used.

#### 3.5.4. Analysis of Cell–Cell and Cell–Substrate Contacts

**Figure 11:**
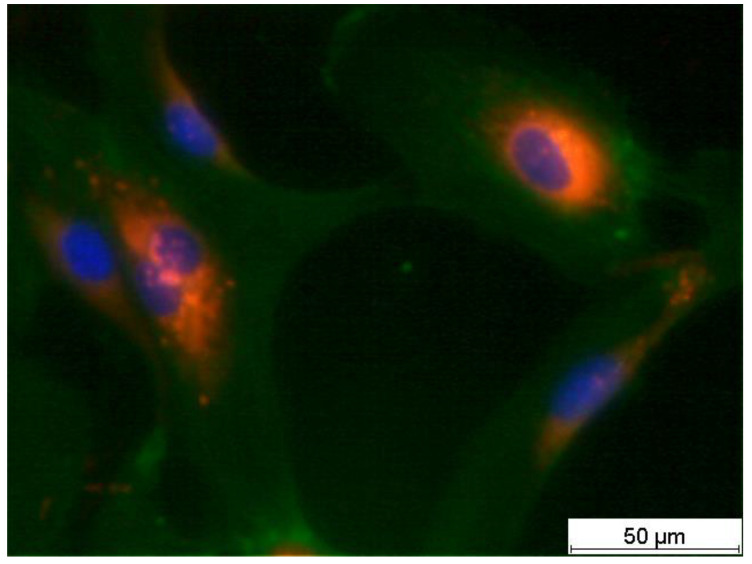
Representative image of paraformaldehyde-fixated HUVECs in culture medium supplemented with 100 µg/mL APE 85 h after seeding (HUVECs were fluorescently stained threefold, the actin cytoskeleton in green, von Willebrand factor in red, and genomic DNA in blue; scale bar: 50 µm).

Microscopic examination of the cells showed that they were endothelial cells (in this case, HUVECs), as expected, based on the red staining of von Willebrand factor (Figure 11). 

**Figure 12:**
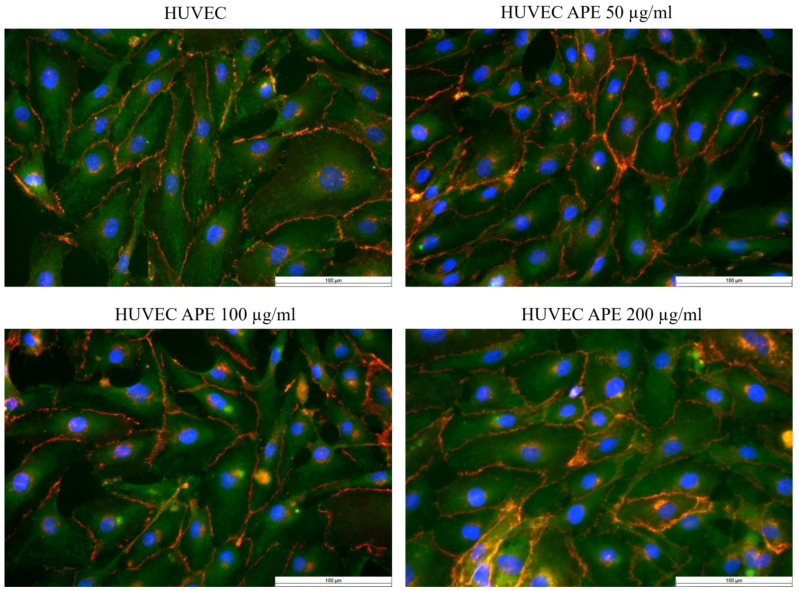
Representative immunofluorescence images of paraformaldehyde-fixated HUVECs in culture medium, HUVECs with 50 µg/mL APE, HUVECs with 100 µg/mL APE, and HUVECs with 200 µg/mL APE 85 h after seeding (40× primary magnification). HUVECs were fluorescently stained threefold: vinculin in green, VE-cadherin in red, and cell nuclei in blue; scale bar: 100 µm.

Figure 11 shows adherent HUVECs after supplementation of the culture medium with 100 µg/mL APE. Here, a beginning regression of stress fibres from the centre of the cells to the edge of the cells was observed as an incipient peripheral filament band, as described for the development of confluent endothelial cell monolayers. The formation of a functionally confluent monolayer with regression of the central stress fibres to the cell edge usually takes up to 9 days (Figure 11 and Figure 12). 

Vinculin and VE-cadherin were also stained. Vinculin is a typical cell marker that is primarily responsible for establishing cell–substrate contacts. Therefore, the distribution and expression of this protein can also be used to infer the attachment of endothelial cells to the underlying substrate. VE-cadherin (vascular endothelial cadherin) is a protein found at contact sites between neighbouring endothelial cells. This protein is therefore essential for the maintenance and control of endothelial cell contacts and thus, among other things, for the control of vascular permeability and leukocyte extravasation.

Forty-eight or eighty-five hours after cell seeding, all HUVECs (with or without APE) showed uniform vinculin expression. Thus, the formation of cell–cell and cell–substrate contacts was unaffected by the addition of APE.

## 4. Discussion

The study revealed that APE at a concentration of 100 µg/mL resulted in a significant increase in adherent HUVECs compared to untreated control cells after 48 h and especially 85 h days of cultivation. Figure 2 clearly shows that the addition of 100 µg/mL APE led to a significant increase in the density of HUVECs already after 48 h (+25%) and even more significantly after 85 h (+52%) of culturing. This confirms previous results from a pilot study [17].

Some of AP’s ingredients are known to possess antioxidant, anti-inflammatory, and antithrombotic properties and can prevent endothelial dysfunction [10,11,12,27]. In particular, C-phycocyanin has been proposed to be a major contributor to these beneficial effects [8,28,29,30]. Consumption of microalga biomass and its derivatives can therefore have a positive effect on the health of consumers by preventing or reducing diseases [31,32,33,34]. It should be noted that the aqueous extract of AP also may contain other substances, such as ɣ-linolenic acid, α-tocopherol, allophycocyanin, phycoerythrin, and other phytochemicals [35,36], which may also contribute to the effects found in this study. 

However, the exact molecular mechanism by which APE achieves the increased HUVEC density remains unclear. One possible reason could be the supplementation of cells with iron from APE. Initial studies showed a differential sensitivity of HUVEC to cell-permeable iron depending on the proliferation status, whereby quiescent endothelial cell viability was not affected [37]. There are studies analysing the iron content in APE [38,39,40]. However, the nature of the iron-binding ligands and in particular the bioavailability of the metal in Arthrospira are still an open question. Whether and how the iron content in spirulina influences the proliferation of endothelial cells, at least in part, is currently unclear. 

After supplementation of the culture medium with APE, significantly more viable HUVECs adhered compared to control cells (see Figure 2, nearly no death cells were visible), the damage to the outer cell membrane was prevented and less LDH was detected in the culture medium compared to the untreated control, while at the same time the metabolic activity was increased, hinting at an increased proliferation of the HUVECs.

The activation or perturbation of the endothelial cells, as previously described as a reaction to altered conditions in the culture milieu [41,42,43,44,45], also occurred here in the first two days after the addition of APE at concentrations of 50 and 200 µg/mL, but was completely prevented at a concentration of 100 µg/mL. Endothelial cells—and many other cells—are involved in the production of IL-6 in response to various stimuli [46,47], e.g., leading to an activation of the immune system. The pattern of the IL-6 release of HUVECs with APE cultivation was very similar to that of prostacyclin. Here also, no increase in IL-6 concentration compared to that in control cells occurred for the APE concentration of 100 µg/mL (see Figure 10).

Interestingly, no TXA2 was detected in the cell culture supernatant of HUVECs cultured with APE. Either TXA2 was not released or, more likely, the amount of TXA2 released was below the detection limit of 15.6–1000.0 pg/mL of the assay used [48], so that the effects of PGI2—the inhibition of platelet activation, the reduction of the risk of thrombosis, vasodilatation, and reduction in vascular smooth muscle cell remodelling and cholesterol uptake [49]—predominated and tipped the balance in favour of prostacyclin, thereby creating an antithrombotic state.

These release responses consistently indicated that there was no inflammatory response of the endothelial cells after adding 100 µg/mL APE to the culture medium. 

When interpreting the results, it should be noted that there may be slight differences depending on the manufacturer of the aqueous AP extract. According to previous experience, all extracts have an effect on endothelial cells, but to different degrees (see, e.g., [50], which must be taken into account when comparing with previous studies).

The homeostasis of physiological tissues is characterised by a low proliferative activity, whereas conditions such as tissue damage induce regeneration and proliferation. HUVECs can shift from a proliferative to an organised state when the in vitro conditions are changed from those favouring low-density proliferation to those supporting high-density survival. Lipps et al. showed that there is a clear correlation between the proliferation state and endothelial function [51]. This suggests that endothelial functions may be differentially expressed during states of high proliferation and vice versa. Thus, in the present study, the reduced synthesis of, e.g., prostacyclin and IL-6 (after 100 µg/mL APE in comparison to 50 or 200 µ/mL APE) could be associated with increased proliferation and thus stronger growth of the endothelial cell monolayer. A decreased cytokine production due to different proliferation rates of HUVECs was also discussed in [52].

The anti-inflammatory effect of APE may also contribute to the improvement of endothelial proliferation. APE has been shown to reduce both TnFα and TGFβ [53,54]. Reduced levels of either TnFα or TGFβ concentration may support the inhibition of adenine dinucleotide phosphate (NADPH) oxidase activation, providing a potent antioxidant effect that could prevent the overproduction of reactive oxygen species and thereby counteracting apoptosis [55,56] and so leading to an increased proliferation. Chu et al. showed that APE significantly reduced apoptosis [55]. The extract may exert its effect by scavenging the free radicals [57] or by reducing the oxidative damage to the cells possibly by reducing the activation of NADPH [Chu]. Both effects could reduce the activation of the apoptotic pathway [58], which could indirectly contribute to a higher HUVEC density.

In addition, an APE-induced increase in endothelial nitric oxide synthase (eNOS) expression has been repeatedly reported [59,60,61,62]. The subsequent improvement in endothelial NO production [63] would then induce a stimulation of endothelial proliferation [64]. Other studies have attributed its antioxidant capacity [55,65,66] to its content of phenolic compounds, such as α-linolenic acid, α-tocopherol, and phycocyanin [8,67]. 

Forty-eight and eighty-five hours after the cultivation of the HUVECs, immunofluorescence staining was performed. In the first step, the cytoskeleton (actin filaments in green) was stained, which was responsible for maintaining the cell shape and contact with the substrate. Secondly, stress fibres were stained, indicating the activation/perturbation of the HUVECs as well as the adaptation of the HUVECs to the external conditions. Thirdly, vinculin as a marker for cell–cell contacts was stained. Two or four days after cell seeding, HUVECs (with or without APE) showed uniform vinculin expression, so that the formation of cell–cell and cell–substrate contacts appeared to be unaffected by the addition of APE. However, there was a significantly faster increase in HUVEC density, probably due to increased HUVEC proliferation, as there were no differences in HUVEC binding to the substrate. This was also evidenced by the increase in metabolic activity.

An endothelial cell-specific marker was used to definitively characterise HUVECs (von Willebrand factor (vWF) in red). In HUVECs supplemented with 100 µg/mL, a slight regression of stress fibres from the centre to the rim of the HUVECs was observed as an incipient peripheral filament band. Such morphological changes in HUVECs were considered typical for the physiological development of confluent endothelial cell monolayers. The formation of a functionally confluent monolayer with complete regression of the central stress fibres to the cell edge usually takes up to 9 days (Figure 11 and Figure 12). 

To date, in vivo toxicological studies of APE have not revealed any toxic effects on kidney, liver, the reproductive system, or body physiology during or after the administration of acute or chronic doses [68,69,70,71,72]. The administration of phycocyanin at the highest doses (from 0.25 up to 5.0 g/kg body weight (*w*/*w*)) did not cause any significant signs of toxicity or mortality in animals [73]. A United States Pharmacopoeia safety assessment—based on a PUBMED literature review from 1966 to 2009 and adverse event reports from the United States Food and Drug Administration (FDA)—from the FDA concluded that AP has a Class A safety [74]. The World Health Organization recommended that the total daily consumption of nucleic acids by humans should not exceed 4 g [75], which corresponds to approximately 100 g of microalgal biomass.

## 5. Conclusions

In conclusion, an increase in proliferation of in vitro HUVECs was induced by an aqueous extract of *Arthrospira platensis* in this prospective, placebo-controlled, sample-size-estimated study. The highest proliferation was achieved after the addition of 100 µg/mL APE. This was consistently confirmed by two independent optical experiments in cell cultures 48 h and 85 h after seeding, as well as by a continuous measuring of the impedance-based method (xCELLigence) and an indirect method (MTS test). At this concentration, the activation or dysfunction of HUVECs was completely prevented, as confirmed by measurements of prostacyclin and interleukin-6 levels.

Since neither cell–cell (VE-cadherin) nor cell–substrate (vinculin) binding was affected by APE, it is reasonable to assume that the increase in endothelial density was due to the proliferation of HUVECs.

In summary, it can be stated that an increase in endothelial cell proliferation can be of great benefit for the regeneration of damaged vessel walls, e.g., after catheter intervention or surgery, or for the restoration of vessel integrity and thereby to the prevention of thrombotic events. Increased endothelial cell proliferation can further accelerate wound healing and contribute to the treatment of ischaemic and chronic vascular diseases.

## Figures and Tables

**Table 1:** Cell indices (CIs) for the three placebo-controlled test series using the xCelligence system. Statistical analysis was performed using *t*-test.

Tests	CI after 85 h (Mean ± Standard Deviation)	*p*
Test series 1, n = 8		
HUVECs + culture medium	3.9 ± 0.2	
HUVECs + APE 50 µg/mL + culture medium	3.8 ± 0.2	*p* = 0.6
Test series 2, n = 8		
HUVECs + culture medium	3.2 ± 0.2	
HUVECs + APE 100 µg/mL + culture medium	3.9 ± 0.3	*p* = 0.0054
Test series 3, n = 8		
HUVECs + culture medium	4.3 ± 0.4	
HUVECs + APE 200 µg/mL + culture medium	3.7 ± 0.3	*p* = 0.0001

## Data Availability

Data is contained within the article.
